# Foramen Tympanicum or Foramen of Huschke: A Bioarchaeological Study on Human Skeletons from an Iron Age Cemetery at Tabriz Kabud Mosque Zone

**Published:** 2015-07

**Authors:** Jafar Rezaian, Mohammad Reza Namavar, Hamed Vahdati Nasab, Ali Reza Hojabri Nobari, Ali Abedollahi

**Affiliations:** 1 Department of Anatomical Sciences, School of Medicine, Lorestan University of Medical Sciences, Khoramabad, Iran; 2 Department of Anatomical Sciences, School of Medicine, Shiraz University of Medical Sciences, Shiraz, Iran;; 3 Histomorphometry and Stereology Research Center, School of Medicine, Shiraz University of Medical Sciences, Shiraz, Iran;; 4 Department of Archeology, Faculty of Humanities, Tarbiat Modares University, Tehran, Iran;; 5 Department of Anatomical Sciences, School of Medicine, Tabriz University of Medical Sciences, Tabriz, Iran

**Keywords:** Foramen tympanicum, Bioarcheological study, Iran

## Abstract

The foramen tympanicum is an anatomical variation that is created in the tympanic plate of temporal bone during the first year of life. The tympanic plate grows and foramen tympanicum is gradually closed by about the fifth postnatal year. However, due to a defect in normal ossification, foramen tympanicum sporadically remains throughout life. The construction of a shopping center in Tabriz, northwest of Iran, led to the discovery of an Iron Age cemetery (1500-500 BC). Several tombs have been uncovered below one meter of sterile soil so far and a thick level of architectural debris from the medieval city has been discovered. Up to now, no bioarchaeological data has been gathered about the burials in this area. Thus, the present study aimed to evaluate the prevalence of foramen tympanicum in this area. In this study, 45 skeletons were studied and the prevalence of this foramen was about 4.4% bilaterally. We also reported on two babies with fused and un-fused squamotympanic fissure. The persistence of this foramen is a possible risk factor for otologic complications after arthroscopy of the temporomandibular joint and salivary gland fistula through this foramen. The closure of this foramen could be also used for age estimation in sub-adult individuals. The incidence of this trait in this study was similar to other available studies on modern skeletons.

## Introduction

The Foramen Tympanicum (FT), or the foramen of Huschke, is a non-metric trait in the tympanic portion of the temporal bone and may remain throughout life.^1^^,^^2^ It was first described by Emil Huschke in 1889. In most cases, FT is located on the inferior wall (floor) of the external acoustic canal on the tympanic part of the temporal bone, but sometimes it appears at the apex of squamotympanic and petrotympanic fissures. It establishes a connection between the external acoustic canal and the mandibular fossa.^3^^,^^4^ The tympanic ring appears first at the embryonic period from mesenchymal tissue lying between the first and second branchial arches.^5^^,^^6^ Multiple ossiﬁcation centers join together to form an incomplete ring, opened above at the tympanic incisures. At birth, there is usually localized fusion of the opened ends to the squamous portion of the temporal bone. The inner surface is grooved by the tympanic sulcus for the attachment of the tympanic membrane, and two bulges, the tympanic tubercles, may be evident. The anterior tympanic tubercle lies near the anterior opened end, and the posterior tubercle lies about halfway down the posterior limb of the ring.^7^

Iron Age (about 1800 to 500 years BC) has a high value through the base fabric of the Iranian plateau. By overcoming the climate harshness and following the victory of the nomadic people, the first cultural foundation of historic Iran begun in this era. From historical and anthropological points of view, the Northwest of Iran has a high value. Cultures of Neolithic, Chalcolithic, Bronze, and Iron ages can be found in this region that implies its enriched and antiquity history.^8^ Tabriz is the most famous city of this region (latitude of 38° 4′ N and longitude of 46°18′ E; 1348-1561 m above the sea level).

To the best of our knowledge, no physical anthropological study has been conducted regarding this period in this region of Iran. Therefore, studies are being performed on skeletons from this area and period. The present study aims to report FT in these skeletons. The anatomical knowledge of FT may be beneficial for anatomists, anthropologists, radiologists, and surgeons. 

## Materials and Methods

In 1997, during the construction of a shopping center adjacent to the Blue Mosque (Masjed-e-Kabud) in Tabriz, the remains of a pre-historical cemetery were discovered.^9^ The consequent archaeological excavations uncovered several graves dating back to the Iron Age. This site has witnessed four seasons of archaeological excavations starting in 2000.^10^ All remains of the skeletons, such as teeth and bones, were conserved in the site museum. The most important remains of the Blue Mosque site are graves belonging to the first millennium BC.^11^ The present cases are based on the study of 45 skeletons (27 adult males, 13 adult females, and 5 children) that are located in the Tabriz site museum. The Ethics Committee of Shiraz University of Medical Sciences has approved the procedures for this research project.

**Figure 1:**
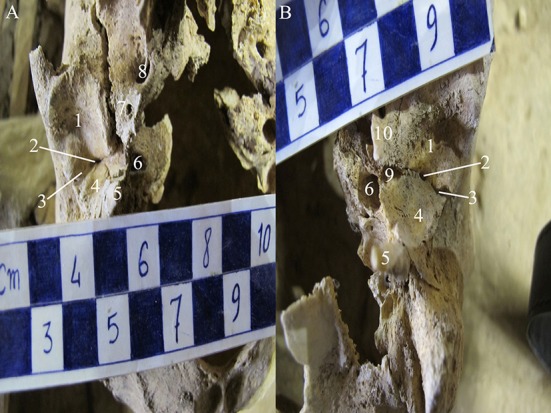
Photographs show the base of the skull. A) Right side, B) Left side (burial number: MK-79-18). 1: Anterior portion of mandibular fossa; 2: Foramen tympanicum; 3: Squamotympanic fissure, 4: Posterior portion of mandibular fossa; 5: Styloid process; 6: Carotid canal; 7: Foramen spinosum; 8: Foramen ovale; 9: Pharyngotympanic tube; 10: Spine of sphenoid

The first skull belonged to a female aged about 25-30 years old (burial number: MK-79-18). The age was estimated based on tooth-wear patterns and Cranial Suture Closure.^12^ Foramina tympanicum were seen in the bilateral side of the skull. They were located at the apex (medial end) of squamotympanic fissure. The foramina were oval and oblong (figure 1). Besides, FT in the left side (figure 1B) was larger than that on the right side (figure 1A).

**Figure 2:**
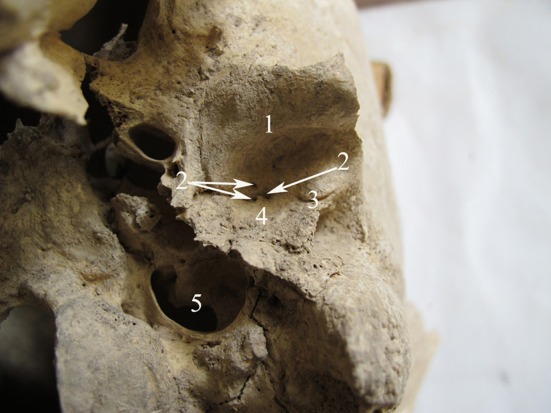
Photograph shows the base of the skull from burial number MK-82-22. 1: Anterior portion of mandibular fossa; 2: Foramen tympanicum; 3: Squamotympanic fissure; 4: Posterior portion of mandibular fossa; 5: Jugular foramen

The second case was a male of about 30-35 years of age (burial number: MK-82-22). Age estimation was based on tooth-wear patterns and Cranial Suture Closure.^12^ Three foramina tympanicum were only seen on the left side of the skull. Two foramina were located at the apex (medial end) of squamotympanic fissure and one on the anterior portion of the mandibular fossa. The foramina were round (figure 2).

**Figure 3:**
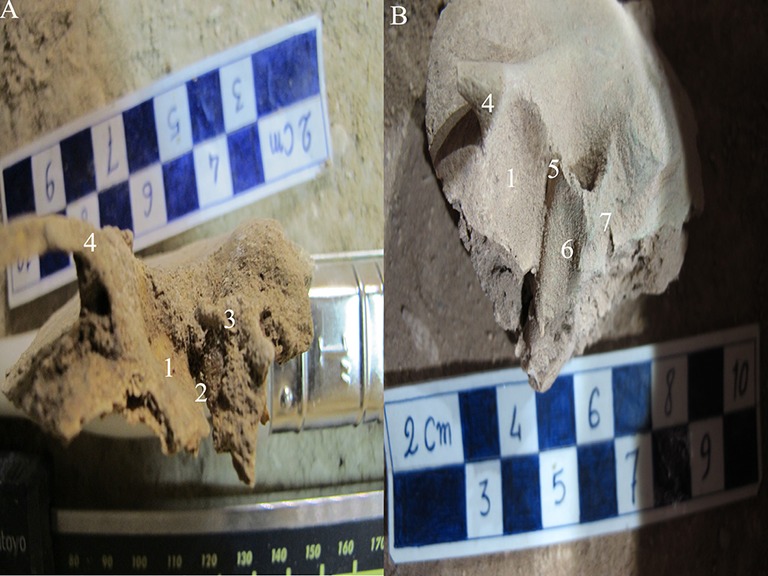
Left temporal bone in a baby of 2-3 years old (A) (burial number: MK-82-2) in comparison with the temporal bone of an almost six-year old child (B) (burial number MK-82-10). 1: Mandibular fossa (anterior portion); 2: Unfused squamotympanic fissure; 3: Scutum and mastoid process; 4: Zygomatic process; 5: Fused squamotympanic fissure; 6: Mandibular fossa (posterior portion); 7: Mastoid process

The following cases are not variations; however, they are introduced here to compare the fused (figure 3A) and un-fused (figure 3B) squamotympanic fissure. Figure 3A shows a baby (burial number: MK-82-2) that might be about 2-3 years old with perfect teeth eruption.^12^ However, there was no attrition on his/her teeth. His/her skeleton was buried in the east–west axis and, therefore, it was accessible only in the left temporal bone. Figure 3B shows a fused squamotympanic fissure in an almost six-year old child skull (burial number: MK-82-10).

## Discussion

The temporal bones form a part of the skull base and the lateral wall of the middle cranial fossa. In the embryo, the temporal bone consists of five separate sections: periodic, squamous, tympanic, stylohyal, and tympanohyal. However, in adults, each bone is a compound structure composed of four portions: the petromastoid, squamous, tympanic, and styloid process.^13^ The tympanic ring is usually recognizable at the middle of the fetal period; however, the closure will start later. Tympanic ring has two anterior and posterior horns.^7^ At the time of birth, the ring is slightly sturdier and the posterior portion will fuse to the pointed end of the scutum. Posterior inferior to the root of the zygomatic process, in the prenatal part of squama, is the scutum. Here, there is a pointed bulge at the petrotympanic fissure.^5^ During the first year of life, anterior and posterior tympanic horns enlarge, grow, and fuse. In some cases, this ring is partially closed. Its second opening is called foramen tympanicum or foramen of Huschke. It is below the osseous portion of the external auditory meatus. It is obvious that squamotympanic fissure is open in the initial years after birth (figure 3A) and will be completely fused in the fifth year of life (figure 3B). In some cases, the foramen may remain in the adult skull. The prevalence of this foramen in this study was about 4.4% bilaterally. However, its prevalence has been reported to range from 0.6% to 46% in different studies.^13^ Some studies have reported the prevalence of this foramen on dried skulls to be about 7%,^9^ while others have used CT and reported the prevalence at about 4.6%,^1^ 11.3%,^14^ and 17.6%.^15^ Such wide range of prevalence can be attributed to the race, method, samples, and population. The present study was conducted on a uniform and prehistoric population in Iran. The closure of this foramen could be used for age estimation in sub-adult individuals.^7^ Also, the persistence of this foramen is a possible risk factor for otologic complications after arthroscopy of the temporomandibular joint, herniation of the temporomandibular joint into the external acoustic meatus, tumor metastasis into the cranial cavity, and salivary gland fistula through this foramen.^13^^,^^16^

## Conclusion

The prevalence of FT in the present study was about 4.4% that is important for specialists in anthropology, ear, temporomandibular joint, and the adjacent regions. 
